# Radiomics-Based Machine Learning for the Detection of Myometrial Invasion in Endometrial Cancer: Systematic Review and Meta-Analysis

**DOI:** 10.2196/78809

**Published:** 2025-11-27

**Authors:** Wanhao Jiang, Huali Wang, Yumeng Cui

**Affiliations:** 1 Dalian Medical University Dalian China; 2 Dalian Women and Children's Medical Center (Group) Dalian China

**Keywords:** endometrial cancer, machine learning, myometrial invasion, magnetic resonance radiomics, artificial intelligence, AI

## Abstract

**Background:**

Preoperative endometrial cancer (EC) diagnosis often depends on radiologists’ expertise, which introduces subjectivity. Recent studies have explored radiomics-based machine learning (ML) models for detecting myometrial invasion (MI), but a comprehensive evaluation of their diagnostic performance is lacking. Therefore, our study systematically assessed the diagnostic performance of radiomics-based ML approaches for identifying MI in EC, thereby providing evidence to guide the development or improvement of noninvasive diagnostic tools.

**Objective:**

This study aims to systematically assess the diagnostic performance of radiomics-based ML approaches for identifying MI in EC and compare the diagnostic efficacy of conventional ML (CML) and deep learning (DL) models based on differences in data processing methods via subgroup analyses, thereby providing evidence to guide the development or improvement of noninvasive diagnostic tools.

**Methods:**

PubMed, Cochrane Library, Embase, and Web of Science were searched through November 26, 2024, for studies evaluating radiomics-based ML for detecting MI in patients with EC. Study quality was appraised using the radiomics quality score. Pooled diagnostic metrics were estimated using a bivariate random-effects model. Subgroup analyses compared CML and DL models.

**Results:**

We included 19 studies comprising 4373 patients with EC. Of these 19 studies, 18 (95%) used magnetic resonance imaging–based radiomics, and 1 (5%) used ultrasound imaging. The pooled estimates from the meta-analysis demonstrated a sensitivity of 0.79 (95% CI 0.73-0.83), a specificity of 0.83 (95% CI 0.79-0.86), a positive likelihood ratio (PLR) of 4.5 (95% CI 3.5-5.8), a negative likelihood ratio (NLR) of 0.26 (95% CI 0.20-0.34), a diagnostic odds ratio (DOR) of 17 (95% CI 11-28), and an area under the summary receiver operating characteristic curve (AUSROC) of 0.89 (95% CI 0.00-1.00). Subgroup analyses revealed that the DL models achieved a sensitivity of 0.81 (95% CI 0.71-0.88) and a specificity of 0.86 (95% CI 0.76-0.92). The PLR, NLR, DOR, and AUSROC were 5.6 (95% CI 3.2-9.8), 0.22 (95% CI 0.14-0.36), 25 (95% CI 10-64), and 0.89 (95% CI 0.00-1.00), respectively. By contrast, the CML models exhibited a sensitivity of 0.77 (95% CI 0.69-0.83) and a specificity of 0.81 (95% CI 0.77-0.85). The PLR, NLR, DOR, and AUSROC were 4.1 (95% CI 3.2-5.4), 0.28 (95% CI 0.20-0.39), 15 (95% CI 9-25), and 0.86 (95% CI 0.00-1.00), respectively.

**Conclusions:**

Radiomics-based ML shows strong potential for noninvasive prediction of MI in EC, with DL outperforming CML. However, current evidence is limited and relies mainly on internal validation. Larger-scale, multicenter studies are needed to establish robust artificial intelligence–based diagnostic tools.

**Trial Registration:**

PROSPERO CRD420250625797; https://www.crd.york.ac.uk/PROSPERO/view/CRD420250625797

## Introduction

Endometrial cancer (EC) is one of the most common gynecologic malignancies worldwide. GLOBOCAN 2020 reported nearly 417,000 new cases (approximately 2% of all cancers) and approximately 97,000 deaths or 1% of cancer-related mortality [1]. The incidence of EC is expected to rise over the next decade [2,3]. Notably, its standardized incidence in low- or middle-income countries in Asia has been increasing annually, approaching that of high-income countries [4].

Accurate staging based on the International Federation of Gynecology and Obstetrics (FIGO) system is essential for guiding surgical decisions [5,6]. Overtreatment can cause unnecessary complications, while undertreatment increases the risk of disease progression [7].

Among the FIGO parameters, the depth of myometrial invasion (MI) is particularly critical. Tumors with MI of ≥50% (FIGO stage IB) require more extensive surgery, including pelvic lymphadenectomy [8]. However, preoperative assessment of MI predominantly relies on transvaginal ultrasonography and magnetic resonance imaging (MRI) [9], with diagnosis precision highly dependent on the radiologist’s expertise [10-12]. Studies have highlighted concerning levels of interobserver variability, with Cohen κ values ranging from 0.43 to 0.67 across institutions [13].

Advances in artificial intelligence (AI) offer promising solutions [14], and interest in EC applications is growing [15]. Li et al [11] leveraged 2 machine learning (ML) models—an ensemble classifier with automated hyperparameter optimization and least absolute shrinkage and selection operator regression—integrating clinical and MRI features to achieve an area under the receiver operating characteristic curve (AUROC) of 0.97 for histological prediction. This approach reduces reliance on intraoperative frozen section pathology and demonstrates the potential of ML for noninvasive preoperative evaluation. Similarly, Zhu et al [16] first quantified MI using the geometric relationship between the uterus and the tumor, laying a more rational basis for region segmentation and texture feature extraction. These findings further exemplify the superiority of ML in radiographic feature analysis. Several studies have also explored radiomics-based ML models for detecting MI in EC [17,18]. However, most studies are limited by small sample sizes and lack systematic validation. In the systematic review conducted by Di Donato et al [19], only 5 studies specifically addressed MI, highlighting the scarcity of robust evidence. He et al [20] also reported a few MI-focused studies but did not evaluate the influence of different ML models on outcomes. At present, no reliable evidence exists to verify the effectiveness of radiomics-based ML models in detecting MI in EC. To bridge this gap, we conducted a systematic review and meta-analysis to evaluate the diagnostic performance of these models in identifying deep MI in EC. Our findings aim to support the development of reliable, noninvasive diagnostic tools for preoperative assessment.

## Methods

### Study Registration

Our study followed the PRISMA (Preferred Reporting Items for Systematic Reviews and Meta-Analyses) guidelines. The protocol was registered on PROSPERO (CRD420250625797).

### Eligibility Criteria

#### Inclusion Criteria

The inclusion criteria were as follows: (1) participants were patients diagnosed with EC; (2) study designs were case-control, cohort, or cross-sectional studies; (3) the study developed a complete ML model for predicting MI in EC; and (4) the study was published in English.

#### Exclusion Criteria

The exclusion criteria were as follows: (1) unpublished conference abstracts; (2) studies that conducted only risk factor analyses without developing a complete ML model; (3) studies that lacked key model performance evaluation metrics, such as AUROC values, C-statistics, sensitivity, specificity, accuracy, precision, confusion matrices, *F*_1_-scores, or calibration curves; and (4) studies that performed only univariate predictive accuracy analyses.

### Data Sources and Search Strategy

PubMed, Cochrane Library, Embase, and Web of Science were comprehensively searched. The final search was conducted on November 26, 2024. Medical Subject Headings terms and free-text keywords were used, with no restrictions on location. The search strategy is detailed in Table S1 in Multimedia Appendix 1.

### Study Selection and Data Extraction

The identified articles were uploaded to EndNote (Clarivate) for reference management. After duplicate removal, titles and abstracts were screened to identify potentially eligible studies. Full texts were subsequently retrieved and assessed according to the eligibility criteria. Before data extraction, a standardized form was developed, capturing the following information: first author; publication year; study setting; study design; number of MI cases and training cases with MI; total number of cases and validation cases; model type; variables used for modeling; AUROC values; number of true positives, false positives, true negatives, and false negatives; sensitivity; specificity; precision; and overall accuracy. Study selection and data extraction were conducted independently by 2 reviewers, with disagreements resolved through discussion or consultation with a third reviewer.

### Study Quality Assessment

The radiomics quality score (RQS) was used to assess study quality and risk of bias. This tool consists of 16 items (maximum score: 36) covering domains such as image protocol quality, multiple segmentations, phantom study on all scanners, imaging at multiple time points, feature reduction or adjustment for multiple testing, multivariable analysis with nonradiomic features, detection and discussion of biological correlates, cutoff analyses, discrimination statistics, calibration statistics, prospective study registration in a trial database, validation, comparison to gold standard, potential clinical utility, cost-effectiveness analysis, and open science and data sharing. Two investigators independently conducted the RQS assessment, with discrepancies resolved through discussion or adjudication by a third reviewer.

### Synthesis Methods

The meta-analysis of diagnostic performance was conducted using a bivariate mixed-effects model applied to 2×2 contingency tables from validation datasets. This model accounts for both fixed and random effects while incorporating the intrinsic relationship between sensitivity and specificity. For studies without explicit 2×2 data, contingency tables were reconstructed using reported sensitivity, specificity, precision, and accuracy (refer to the following formula):



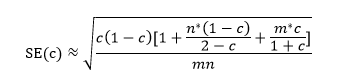



where *c* denotes the C-statistic, *n* denotes the number of observed events, and *m* denotes the total sample size.

The model provided pooled estimates of sensitivity, specificity, positive likelihood ratio (PLR), negative likelihood ratio (NLR), diagnostic odds ratio (DOR), and the area under the summary receiver operating characteristic curve (AUSROC) with corresponding 95% CIs. Publication bias was assessed using the Deeks funnel plot, and clinical applicability was evaluated through the Fagan nomogram. Subgroup analyses were carried out according to model type. Statistical significance was defined as *P*<.05.

## Results

### Study Selection

**Figure 1:**
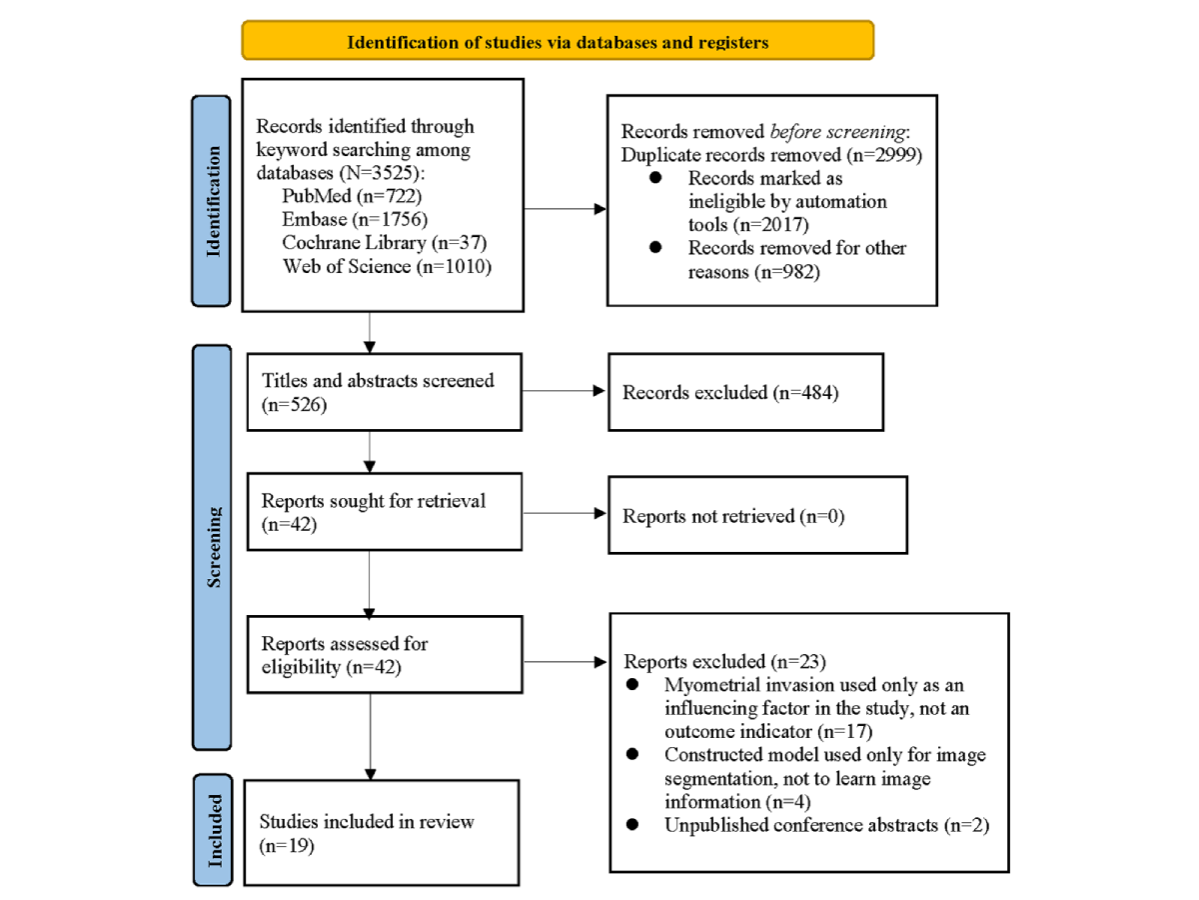
Flow diagram of study selection.

We retrieved 3525 records through a comprehensive database search, of which 912 (26%) duplicates and 2087 (59%) studies irrelevant to the subject of our investigation were removed. The titles and abstracts of the remaining 526 studies were screened, and 25 (5%) studies containing the keywords “EC” and “MI” were shortlisted. After a full-text review, 6 (24%) of the 25 studies were excluded for not aligning with the research objectives, leaving 19 (76%) studies for inclusion in the meta-analysis [11,13,16-18,21-34] (Figure 1).

### Study Characteristics

The eligible studies, which were published between 2019 and 2024, comprised 4373 patients with EC, of whom 1221 (28%) had identifiable MI. All studies defined MI as an infiltration depth greater than 50%. Of the 19 studies, 4 (21%) used multicenter cohorts for validation, while the remaining 15 (79%) relied on randomly selected samples. Regarding modeling approaches, 5 (26%) of the 19 studies applied deep learning (DL) algorithms, and 14 (74%) used ML techniques. Of the 19 studies, 17 (89%) used MRI-based radiomic features, 1 (5%) incorporated apparent diffusion coefficient parameters, and 1 (5%) used ultrasound radiomic features as input variables (Table 1).

**Table 1:** Baseline characteristics of the included studies (n=19).

Study; country	Study setting	Definition of MI^a^	MI cases, n	Total cases, n	MI cases in the training set, n	Total cases in the training set, n	Validation set generation method	MI cases in the validation set, n	Total cases in the validation set, n	Imaging modality
Chen et al [33], 2020; China	Single center	MI >50%	99	530	57	313	Random sampling	Dataset 1: 24; dataset 2: 18	Dataset 1 :79; dataset 2: 138	MRI^b^
Dong et al [32], 2020; China	Single center	MI >50%	19	72	—^c^	24	Random sampling	—	48	MRI
Han et al [13], 2020; China	Single center	MI >50%	44	163	—	—	Random sampling	—	—	MRI
Jiang et al [25], 2023; China	Single center	MI >50%	35	158	24	110	Random sampling	11	48	MRI
Li et al [11], 2023; United Kingdom	Multicenter	FIGO^d^ IB	—	495	292	413	Random sampling	14	82	MRI
Liu et al [21], 2024; China	Multicenter	MI >50%	206	609 I:1289	144	509	Internal validation and external validation	Dataset 1: 31; dataset 2: 31	Dataset 1: 95; dataset 2: 95	Ultrasound
Mao et al [18], 2023; China	Single center	MI >50%	65	134	45	96	Random sampling	20	38	MRI
Qin et al [27], 2022; China	Single center	FIGO IB	22	348	12	243	Random sampling	10	105	MRI
Rodríguez-Ortega et al [31], 2021; Spain	Single center	MI >50%	62	143	46	107	Random sampling	16	36	MRI
Stanzione et al [30], 2021; Italy	Single center	MI >50%	17	54	14	43	Random sampling	3	11	MRI
Xiong et al [23], 2023; China	Single center	MI >50%	71	154	50	108	Random sampling	21	46	MRI
Zhu et al [16], 2021; China	Single center	MI >50%	19	79	—	—	Multicenter	—	—	MRI
Zhao et al [26], 2022; China	Multicenter	MI >50%	69	163	46	107	Multicenter	23	56	MRI
Fang et al [17], 2024; China	Single center	MI >50%	79	198	55	138	Random sampling	24	60	MRI
Ma et al [35], 2023; China	Single center	MI >50%	95	292	49	204	Random sampling	31	88	MRI
Miccò et al [34], 2022; Switzerland	Multicenter	MI >50%	60	124	50	98	Multicenter	10	26	MRI
Lefebvre et al [36], 2022; Canada	Multicenter	MI >50%	79	157	43	94	Multicenter	36	63	MRI
Zhang et al [29], 2021; China	Single center	MI >50%	53	210	53	210	—	—	—	MRI
Otani et al [28], 2022; Japan	Single center	MI >50%	127	200	95	150	Random sampling	32	50	MRI

^a^MI: myometrial invasion.

^b^MRI: magnetic resonance imaging.

^c^Not applicable.

^d^FIGO: International Federation of Gynecology and Obstetrics.

### Study Quality Assessment

Several domains were not fully addressed in the included studies, resulting in partial scores in the quality assessment. Factors such as the high cost of MRI examinations and poor patient adherence to repeated scans limited reproducibility. None of the studies collected imaging at multiple time points. Most (14/19, 74%) were single-center studies, and repeated scans on different devices were rarely feasible due to equipment costs. Device-related differences were not assessed in any study, resulting in zero points for interscanner variability. Furthermore, none of the studies reported prospective protocols or registration in publicly accessible databases. The vast majority of the studies (18/19, 95%) did not examine vendor-specific dependencies or biological correlates. Potential clinical utility was not assessed in 16 (84%) of the 19 studies, and calibration statistics were missing in 15 (79%) studies, while 12 (63%) did not perform multivariable analyses incorporating nonradiomic clinical features. Of the 19 studies, 14 (74%) omitted threshold effect analyses, and 4 (21%) did not disclose open science practices or share datasets. The final quality assessment based on the RQS is shown in Table 2.

The RQS assessment also highlighted substantial deficiencies across the included studies. Many of the studies (n/N, %) lacked preliminary experiments, making certain items difficult to score. The included studies all lacked preliminary experiments, making it difficult to score certain items. Differences in imaging protocols across devices were generally not addressed. Notably, RQS scoring for validation methods ranges from −5 to +5 points, with negative points assigned when no validation is performed. Multicenter studies receive higher scores, but such studies are scarce due to data barriers, contributing to lower overall RQS scores.

**Table 2:** Quality assessment of the included studies (n=19) based on the radiomics quality score (RQS; maximum score: 36).

Study	RQS item																	Total score		Proportion of maximum RQS (%)
	1^a^	2^b^	3^c^	4^d^	5^e^	6^f^	7^g^	8^h^	9^i^	10^j^	11^k^	12^l^	13^m^	14^n^	15^o^	16^p^				
Chen et al [33], 2020	1	1	0	0	−1	0	0	0	1	0	0	2	0	0	0	2	6		17	
Dong et al [32], 2020	1	1	0	0	0	0	0	0	1	0	0	2	0	2	0	0	7		19	
Han et al [13], 2020	1	1	0	0	0	0	0	0	2	0	0	2	0	0	0	1	7		19	
Jiang et al [25], 2023	1	1	0	0	0	0	0	1	1	0	0	2	0	0	0	1	7		19	
Li et al [11], 2023	1	1	0	0	0	1	0	1	1	0	0	3	0	0	0	2	10		28	
Liu et al [21], 2024	1	1	1	0	0	1	0	0	1	0	0	4	0	2	0	0	11		31	
Mao et al [18], 2023	1	1	0	0	0	0	0	0	1	0	0	2	0	0	0	1	6		17	
Qin et al [27], 2022	1	1	0	0	0	1	0	0	1	0	0	2	0	0	0	0	6		17	
Rodríguez-Ortega et al [31], 2021	1	1	0	0	0	0	0	0	1	0	0	2	0	0	0	1	6		17	
Stanzione et al [30], 2021	1	1	0	0	0	0	0	0	2	0	0	2	0	0	0	1	7		19	
Xiong et al [23], 2023	1	1	0	0	0	0	0	1	1	0	0	2	0	0	0	2	8		22	
Zhu et al [16], 2021	1	1	0	0	0	0	0	0	1	0	0	2	0	0	0	2	7		19	
Zhao et al [26], 2022	1	1	0	0	0	1	0	1	2	1	0	3	0	2	0	1	13		36	
Fang et al [17], 2024	1	1	0	0	0	0	0	0	2	1	0	2	0	0	0	1	8		22	
Ma et al [35], 2023	1	1	0	0	0	1	1	1	2	1	0	2	1	0	0	0	11		31	
Miccò et al [34], 2022	1	1	0	0	0	1	0	0	1	0	0	3	0	0	0	1	8		22	
Lefebvre et al [36], 2022	1	1	0	0	0	0	0	0	1	0	0	3	0	0	0	1	7		19	
Zhang et al [29], 2021	1	1	0	0	1	0	0	0	1	2	0	2	0	0	0	2	10		28	
Otani et al [28], 2022	1	1	0	0	0	1	0	0	1	0	0	2	0	0	0	1	7		19	

^a^Image protocol quality.

^b^Multiple segmentations.

^c^Phantom study on all scanners.

^d^Imaging at multiple time points.

^e^Feature reduction or adjustment for multiple testing.

^f^Multivariable analysis with nonradiomic features.

^g^Detection and discussion of biological correlates.

^h^Cutoff analyses.

^i^Discrimination statistics.

^j^Calibration statistics.

^k^Prospective study registration in a trial database.

^l^Validation.

^m^Comparison to gold standard.

^n^Potential clinical utility.

^o^Cost-effectiveness analysis.

^p^Open science and data sharing.

### Meta-Analysis

#### All Results

**Figure 2:**
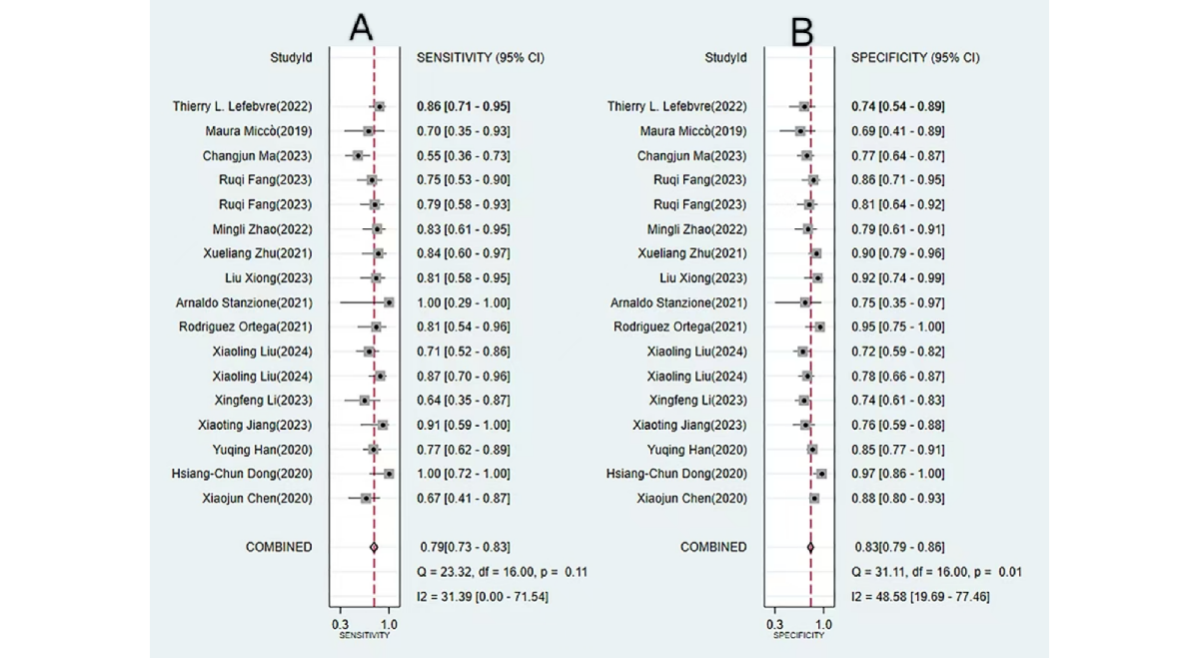
Forest plots of pooled sensitivity and specificity for machine learning–based prediction of myometrial invasion in endometrial cancer. Both plots demonstrate minimal heterogeneity, indicating a high degree of consistency across the included studies. (A) Forest plot depicting the pooled sensitivity from the meta-analysis. (B) Forest plot depicting the pooled specificity. [11, 13, 16, 17, 21, 23, 25, 26, 30-36].

**Figure 3:**
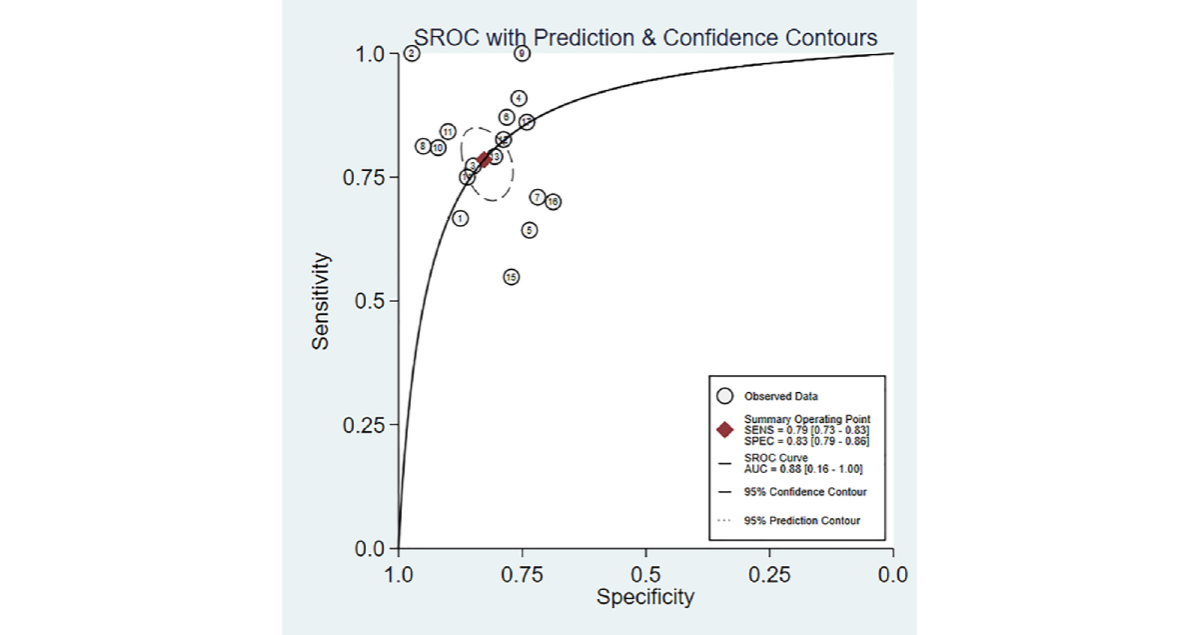
Area under the summary receiver operating characteristic curve (AUSROC) with prediction and confidence contours for machine learning–based prediction of myometrial invasion in endometrial cancer. AUSROC values fitted from the study estimates indicate no threshold effect.

We analyzed 17 diagnostic contingency tables to assess the performance of ML models in identifying MI in EC. The pooled sensitivity and specificity were 0.79 (95% CI 0.73-0.83; *I*²=31.39%) and 0.83 (95% CI 0.79-0.86; *I*²=48.58%), respectively. The pooled PLR, NLR, DOR, and AUSROC were 4.5 (95% CI 3.5-5.8), 0.26 (95% CI 0.20-0.34), 17 (95% CI 11-28), and 0.89 (95% CI 0.00-1.00), respectively (Figures 2 and 3), indicating low heterogeneity and relatively high predictive accuracy.

The Fagan nomogram (Figure S1 in Multimedia Appendix 1) showed that a positive model prediction corresponded to a 66% posttest probability of MI, while a negative prediction corresponded to a 90% probability of absent invasion. The Deeks funnel plot revealed no significant publication bias (*P*=.31; Figure S2 in Multimedia Appendix 1).

#### DL Results

The diagnostic performance of DL models for detecting MI in EC was evaluated across six 2×2 contingency tables. The pooled sensitivity and specificity were 0.81 (95% CI 0.71-0.88; *I*²=41.7%) and 0.86 (95% CI 0.76-0.92; *I*²=71.9%), respectively. The pooled PLR, NLR, DOR, and AUSROC were 5.6 (95% CI 3.2-9.8), 0.22 (95% CI 0.14-0.36), 25 (95% CI 10-64), and 0.89 (95% CI 0.00-1.00), respectively, indicating comparatively high predictive accuracy (Figures S3 and S4 in Multimedia Appendix 1).

The Fagan nomogram showed that a positive DL prediction corresponded to a 50% posttest probability of true MI, whereas a negative prediction corresponded to an 82% probability of true absence (Figure S5 in Multimedia Appendix 1). The Deeks funnel plot suggested significant publication bias (*P*=.04; Figure S6 in Multimedia Appendix 1).

#### Conventional ML Results

The diagnosis performance of conventional ML (CML) models for detecting MI in EC was assessed using 11 diagnostic 2×2 contingency tables. The pooled sensitivity and specificity were 0.77 (95% CI 0.69-0.83; *I*^2^=33.26%) and 0.81 (95% CI 0.77-0.85; *I*^2^=29.12%), respectively. The PLR, NLR, DOR, and AUSROC were 4.1 (95% CI 3.2-5.4), 0.28 (95% CI 0.20-0.39), 15 (95% CI 9-25), and 0.86 (95% CI 0.00-1.00), respectively, indicating low heterogeneity and relatively favorable predictive accuracy (Figures S7 and S8 in Multimedia Appendix 1).

The Fagan nomogram showed that a positive model prediction corresponded to a 50% posttest probability of true MI, whereas a negative prediction corresponded to an 82% probability of true absence (Figure S9 in Multimedia Appendix 1). The Deeks funnel plot revealed insignificant publication bias (*P*=.79; Figure S10 in Multimedia Appendix 1).

## Discussion

### Key Findings

Current ML approaches for detecting deep MI in EC primarily rely on MRI-based radiomics and demonstrate favorable diagnostic performance. Across all included studies, the pooled sensitivity and specificity were 0.79 (95% CI 0.73-0.83) and 0.83 (95% CI 0.79-0.86), respectively. DL models achieved a sensitivity of 0.81 (95% CI 0.71-0.88) and a specificity of 0.86 (95% CI 0.76-0.92), whereas CML models achieved a sensitivity of 0.77 (95% CI 0.69-0.83) and a specificity of 0.81 (95% CI 0.77-0.85).

### Comparison With Previous Studies

Previous studies have also explored the use of MRI-based radiomics for assessing MI in EC. Di Donato et al [19] reviewed MRI-based radiomics studies in EC; of 5 studies on MI, 4 were excluded due to missing AUROC values, validation cohorts, or essential diagnostic data. The pooled sensitivity and specificity in the remaining study were 0.743 (95% CI 0.607-0.844) and 0.816 (95% CI 0.740-0.874), respectively, although the limited sample may reduce generalizability. He et al [20] evaluated ML-based radiomics for preoperative EC assessment, encompassing high-grade lesions, lymphovascular invasion, lymph node metastasis, and deep MI. For MI-specific models using radiomic features, the sensitivity and specificity were 0.80 (95% CI 0.74-0.84) and 0.81 (95% CI 0.76-0.86), respectively, but the impact of model differences on performance was not discussed. CML requires manual image segmentation, feature extraction, and model training, making it labor intensive and subject to interoperator variability. By contrast, DL automatically identifies features from imaging data, reducing workload and variability. Future studies should compare CML and DL performance and explore their clinical applicability. Many previous studies were single-center studies with small samples, whereas our meta-analysis included 19 studies from multiple regions, comprising 4373 patients with EC, enhancing the generalizability of ML for MI detection.

ML is a data-driven methodology that enables predictive or classification tasks through model training. Both CML and DL are subfields of ML, but they differ markedly in terms of model architecture and learning paradigms [37]. CML relies on manually extracted clinical features (eg, blood pressure and glucose levels) and uses interpretable models such as support vector machines, random forests, or logistic regression. By contrast, DL automatically extracts high-dimensional imaging features without manual intervention, achieving superior performance with large datasets and complex patterns but exhibiting a “black box” nature [38]. Given the methodological and practical distinctions between CML and DL in clinical research, our study examined their diagnostic performance separately. The pooled sensitivity and specificity indeed differed between the 2 approaches.

### Modeling Variables

Current ML models typically incorporate clinical features, genetic markers, and laboratory parameters; however, this study specifically focused on MRI-based radiomics. Owing to its noninvasive nature and high spatial resolution, MRI remains indispensable for EC diagnosis, MI assessment, recurrence monitoring, and personalized treatment [12]. The combined application of multiparametric MRI, incorporating T2-weighted imaging, diffusion-weighted imaging, and dynamic contrast-enhanced sequences, has further enhanced diagnostic performance. When applied in an integrated fashion, this approach consistently yields sensitivity and specificity exceeding 80%, substantially outperforming any single sequence modality alone [12].

Most studies derive features from manual radiologist segmentation, which is subjective and may result in the loss of critical texture information. By contrast, DL integrates segmentation and feature extraction within the training process, preserving data integrity and improving accuracy. Our study found that DL also outperformed manual radiologist assessment in both sensitivity and specificity.

Selecting an appropriate modeling approach is challenging due to differences between CML and DL. CML requires manual encoding of variables such as age, FIGO stage, and tumor grade; moreover, MRI images must be segmented manually before feature selection and model training. This multistep process risks losing critical texture information. By contrast, DL automates lesion segmentation and predictive modeling, maximizing feature retention and improving accuracy. Nonetheless, CML retains the advantage of interpretability. In clinical contexts, using interpretable variables remains crucial for models that not only predict outcomes but also clarify their association with underlying clinical parameters.

### Challenges in Translating Radiomics Into Clinical Practice

Although MRI-based radiomics shows promise for detecting MI in EC, substantial challenges remain before clinical implementation. DL excels in image recognition and decision-making, but its “black box” nature limits trust and adoption. While inputs and outputs are observable, the internal reasoning remains largely opaque, raising potential ethical concerns. Explainable AI offers a path forward, helping bridge theory and clinical practice; for example, saliency maps can highlight the regions driving model decisions, while simple interpretable models can approximate complex “black box” reasoning in specific contexts. In terms of datasets, most of the included studies (14/19, 74%) adopted a single-center design, with training and validation sets typically generated through internal random splits of a single dataset (eg, 7:3 or 8:2). Although such studies may produce models with favorable performance, the generalizability and clinical applicability of these models remain questionable due to the common absence of external validation using multicenter datasets. This limitation fundamentally undermines the robustness of the pooled results in this review and highlights the challenges faced by ML models trained on single-center data in achieving broader applicability.

### Future Directions for Clinical Translation

The favorable diagnostic accuracy demonstrated by DL and radiomics in assessing MI in EC delineates a clear pathway for their integration into clinical workflows. These models are most likely to be applied as adjunctive tools within the existing preoperative MRI workflow rather than as stand-alone systems; for example, a DL-based automated algorithm could process MRI images in the background and provide radiologists with a quantitative risk score for deep MI. This would compensate for the limitations of subjective visual assessment; reduce interobserver variability; and enhance diagnostic confidence, particularly for less experienced clinicians.

The integration of such models holds promise for addressing critical bottlenecks in current patient care. One important application lies in improving preoperative risk stratification. By accurately identifying patients at high risk for deep MI, lymphovascular space invasion, or lymph node metastasis, these models can assist clinicians by (1) providing objective evidence to support the recommendation of lymphadenectomy, thereby optimizing surgical planning; and (2) potentially reducing the need for invasive diagnostic procedures in patients at low risk. Combining imaging features with clinical indicators (eg, age and BMI) will be crucial for developing robust multivariable prediction models that capture the complexity of clinical decision-making.

To realize this clinical value, future research must shift from mere technical validation to prospective evaluation in real-world clinical settings. Key steps include demonstrating workflow compatibility, assessing the impact on radiologists’ performance and decision-making (eg, through reader studies), and ultimately establishing whether the use of such models translates into improved patient outcomes. Addressing challenges related to model interpretability and generalizability across different MRI scanners will be essential for widespread adoption.

### Strengths and Limitations of the Study

ML provides comprehensive and systematic evidence for assessing deep MI. Our meta-analysis included 4373 patients with EC, thereby enhancing the objectivity of the study. The pooled results revealed high accuracy and specificity, underscoring the potential clinical utility of ML-based approaches. However, several limitations must be acknowledged. First, although analyses based on both CML and DL approaches were carried out, only a small proportion of studies used external validation cohorts, which possibly compromises the application of our findings. Second, this analysis is constrained by the limited volume of available literature. In particular, high-quality studies focusing on DL methods remain relatively scarce, which may partially affect the interpretation of the generalizability of DL models and constitutes another limitation that warrants attention. We anticipate that future validation studies dedicated to DL will provide new evidence that can be incorporated into subsequent analyses, thereby strengthening the robustness of our conclusions. Third, the RQS assessment indicated that the included studies generally scored low. Methodological flaws, such as the lack of external validation and the failure to account for differences among scanning devices, may have led to biased and overly optimistic diagnostic performance reported in the studies. Although the performance metrics we pooled were favorable, they might not fully represent the true performance of these models in clinical practice. Fourth, the vast majority of studies (14/19, 74%) lacked independent external validation, implying that the pooled performance metrics may be overly optimistic and that reproducibility remains unproven. The single-center design of most of the studies (14/19, 74%) and the failure to account for interscanner variability may further affect the interpretation of results. These common limitations reflect not only the shortcomings of individual studies but also broader challenges in EC radiomics, particularly in data sharing, multicenter collaboration, and methodological standardization. Therefore, the universality of these models across different MRI devices, patient populations, and clinical settings remains unverified, which constitutes a key obstacle to the clinical translation of these models. Finally, publication bias was observed in the DL subgroup analysis (Deeks funnel plot: *P*=.04), suggesting that the high performance reported for DL models may be overestimated. This bias may stem from several factors: a publication preference for positive results, whereby studies with superior performance or favorable outcomes are more likely to be published; a tendency toward reporting successful cases involving complex or novel preprocessing pipelines or model architectures; and limited data and code sharing, which restricts reproducibility and makes negative or neutral results less likely to be validated and published.

### Clinical Translation and Digital Health Policy Prospects

The results of this study highlight the significant potential of radiomics-based ML for the preoperative assessment of MI in EC. However, to promote the application of these models in clinical practice, some problems need to be addressed, such as workflow integration, ethical issues, and regulatory requirements and data policies.

#### Workflow Integration

The most feasible application of these ML models is as an auxiliary decision-making tool for radiologists; for example, the models can be integrated into the hospital system to automatically provide quantitative risk scores for MI and visualize decision areas when the physician reviews MRI images. This human-machine collaborative model can not only compensate for the biases of subjective visual assessment, improving diagnostic consistency, but also free physicians from repetitive tasks, allowing them to focus on the analysis of complex cases.

#### Ethical Issues

Ethical issues arising from the opacity of AI models must be addressed. The “black box” nature of DL models makes it difficult to understand their decision-making logic, which severely hinders the establishment of clinical trust and complicates the determination of responsibility in cases of misjudgments. Therefore, integrating interpretable AI models is one of the main directions in the future; for example, techniques such as saliency maps can visually highlight the image features driving model decisions, making the decision-making process transparent to physicians.

#### Regulatory Requirements and Data Policies

Regulatory requirements and data policies form the foundation for large-scale application. Regulatory agencies require strong prospective multicenter trial evidence to approve such AI tools for clinical practice. At the same time, the process of collecting large-scale data for model training must take into account the protection of patient privacy. Reasonable regulation and data policies are necessary for building diagnostic models with strong generalizability, in line with ethics and regulations.

### Conclusions

Radiomics-based ML models (especially DL models) demonstrate strong diagnostic potential for the noninvasive prediction of MI in EC. However, current evidence, mainly derived from single-center, retrospective studies that have only performed internal validation, remains preliminary. Major challenges such as limited generalizability, the “black box” nature of DL, and potential publication bias have hindered the immediate application of radiomics-based ML models in clinical practice. In the future, it is imperative to conduct large-scale, prospective, multicenter external validation studies and integrate these models into clinical workflows. However, before such tools can be adopted in routine clinical practice, it is essential to address their interpretability and demonstrate their practical impact on clinical decision-making and patient prognosis.

## Appendix

Multimedia Appendix 1 Fagan nomogram analysis, Deeks funnel plot for machine learning; forest plot, SROC curve, Fagan nomogram analysis, Deeks funnel plot for deep learning and conventional machine learning; and search strategies.

Multimedia Appendix 2 PRISMA 2020 checklist.

## Abbreviations

AI: artificial intelligence
AUROC: area under the receiver operating characteristic curve
AUSROC: area under the summary receiver operating characteristic curve
DL: deep learning
DOR: diagnostic odds ratio
EC: endometrial cancer
FIGO: International Federation of Gynecology and Obstetrics
MeSH: Medical Subject Headings
MI: myometrial invasion
ML: machine learning
MRI: magnetic resonance imaging
NLR: negative likelihood ratio
PLR: positive likelihood ratio
PRISMA: Preferred Reporting Items for Systematic Reviews and Meta-Analyses
RQS: radiomics quality score

## Data Availability

All data generated or analyzed during this study are included in this paper and its supplementary information files.

## References

[1] Sung H, Ferlay J, Siegel RL, Laversanne M, Soerjomataram I, Jemal A, Bray F (2021). Global cancer statistics 2020: GLOBOCAN estimates of incidence and mortality worldwide for 36 cancers in 185 countries. CA Cancer J Clin.

[2] Gaber C, Meza R, Ruterbusch JJ, Cote ML (2016). Endometrial cancer trends by race and histology in the USA: projecting the number of new cases from 2015 to 2040. J Racial Ethn Health Disparities.

[3] Wang X, Glubb DM, O'Mara TA (2023). Dietary factors and endometrial cancer risk: a Mendelian randomization study. Nutrients.

[4] Katagiri R, Iwasaki M, Abe SK, Islam MR, Rahman MS, Saito E, Merritt MA, Choi JY, Shin A, Sawada N, Tamakoshi A, Koh WP, Sakata R, Tsuji I, Kim J, Nagata C, Park SK, Kweon SS, Shu XO, Gao YT, Tsugane S, Kimura T, Yuan JM, Kanemura S, Lu Y, Sugawara Y, Wada K, Shin MH, Ahsan H, Boffetta P, Chia KS, Matsuo K, Qiao YL, Rothman N, Zheng W, Inoue M, Kang D (2023). Reproductive factors and endometrial cancer risk among women. JAMA Netw Open.

[5] Tenney M, Walker JL (2009). Role of laparoscopic surgery in the management of endometrial cancer. J Natl Compr Canc Netw.

[6] Candiani GB, Belloni C, Maggi R, Colombo G, Frigoli A, Carinelli SG (1990). Evaluation of different surgical approaches in the treatment of endometrial cancer at FIGO stage I. Gynecol Oncol.

[7] Straughn JM, Huh WK, Kelly FJ, Leath CA, Kleinberg MJ, Hyde J, Numnum TM, Zhang Y, Soong SJ, Austin JM, Partridge EE, Kilgore LC, Alvarez RD (2002). Conservative management of stage I endometrial carcinoma after surgical staging. Gynecol Oncol.

[8] Berek JS, Matias-Guiu X, Creutzberg C, Fotopoulou C, Gaffney D, Kehoe S, Lindemann K, Mutch D, Concin N, Endometrial Cancer Staging Subcommittee, FIGO Women's Cancer Committee (2023). FIGO staging of endometrial cancer: 2023. Int J Gynaecol Obstet.

[9] Freeman SJ, Aly AM, Kataoka MY, Addley HC, Reinhold C, Sala E (2012). The revised FIGO staging system for uterine malignancies: implications for MR imaging. Radiographics.

[10] Alcázar JL, Galván R, Albela S, Martinez S, Pahisa J, Jurado M, López-García G (2009). Assessing myometrial infiltration by endometrial cancer: uterine virtual navigation with three-dimensional US. Radiology.

[11] Li X, Dessi M, Marcus D, Russell J, Aboagye EO, Ellis LB, Sheeka A, Park WE, Bharwani N, Ghaem-Maghami S, Rockall AG (2023). Prediction of deep myometrial infiltration, clinical risk category, histological type, and lymphovascular space invasion in women with endometrial cancer based on clinical and T2-weighted MRI radiomic features. Cancers (Basel).

[12] Maheshwari E, Nougaret S, Stein EB, Rauch GM, Hwang KP, Stafford RJ, Klopp AH, Soliman PT, Maturen KE, Rockall AG, Lee SI, Sadowski EA, Venkatesan AM (2022). Update on MRI in evaluation and treatment of endometrial cancer. Radiographics.

[13] Han Y, Xu H, Ming Y, Liu Q, Huang C, Xu J, Zhang J, Li Y (2020). Predicting myometrial invasion in endometrial cancer based on whole-uterine magnetic resonance radiomics. J Cancer Res Ther.

[14] Garg P, Mohanty A, Ramisetty S, Kulkarni P, Horne D, Pisick E, Salgia R, Singhal SS (2023). Artificial intelligence and allied subsets in early detection and preclusion of gynecological cancers. Biochim Biophys Acta Rev Cancer.

[15] Sivajohan B, Elgendi M, Menon C, Allaire C, Yong P, Bedaiwy MA (2022). Clinical use of artificial intelligence in endometriosis: a scoping review. NPJ Digit Med.

[16] Zhu X, Ying J, Yang H, Fu L, Li B, Jiang B (2021). Detection of deep myometrial invasion in endometrial cancer MR imaging based on multi-feature fusion and probabilistic support vector machine ensemble. Comput Biol Med.

[17] Fang R, Lin N, Weng S, Liu K, Chen X, Cao D (2024). Multiparametric MRI radiomics improves preoperative diagnostic performance for local staging in patients with endometrial cancer. Abdom Radiol (NY).

[18] Mao W, Chen C, Gao H, Xiong L, Lin Y (2023). Quantitative evaluation of myometrial infiltration depth ratio for early endometrial cancer based on deep learning. Biomed Signal Process Control.

[19] Di Donato V, Kontopantelis E, Cuccu I, Sgamba L, Golia D'Augè T, Pernazza A, Della Rocca C, Manganaro L, Catalano C, Perniola G, Palaia I, Tomao F, Giannini A, Muzii L, Bogani G (2023). Magnetic resonance imaging-radiomics in endometrial cancer: a systematic review and meta-analysis. Int J Gynecol Cancer.

[20] He J, Liu Y, Li J, Liu S (2024). Accuracy of radiomics in the diagnosis and preoperative high-risk assessment of endometrial cancer: a systematic review and meta-analysis. Front Oncol.

[21] Liu X, Qin X, Luo Q, Qiao J, Xiao W, Zhu Q, Liu J, Zhang C (2024). A transvaginal ultrasound-based deep learning model for the noninvasive diagnosis of myometrial invasion in patients with endometrial cancer: comparison with radiologists. Acad Radiol.

[22] Jang DH, Lee HG, Lee B, Kang S, Kim JH, Kim BG, Kim JW, Kim MH, Chen X, No JH, Lee JM, Kim JH, Watari H, Kim SM, Kim SH, Seong SJ, Jeong DH, Kim YH (2024). Prediction of final pathology depending on preoperative myometrial invasion and grade assessment in low-risk endometrial cancer patients: a Korean Gynecologic Oncology Group ancillary study. PLoS One.

[23] Xiong L, Chen C, Lin Y, Mao W, Song Z (2023). A computer-aided determining method for the myometrial infiltration depth of early endometrial cancer on MRI images. Biomed Eng Online.

[24] Wang Y, Bi Q, Deng Y, Yang Z, Song Y, Wu Y, Wu K (2023). Development and validation of an MRI-based radiomics nomogram for assessing deep myometrial invasion in early stage endometrial adenocarcinoma. Acad Radiol.

[25] Jiang X, Song J, Zhang A, Cheng W, Duan S, Liu X, Chen T (2023). Preoperative assessment of MRI-invisible early-stage endometrial cancer with MRI-based radiomics analysis. J Magn Reson Imaging.

[26] Zhao M, Wen F, Shi J, Song J, Zhao J, Song Q, Lai Q, Luo Y, Yu T, Jiang X, Jiang W, Dong Y (2022). MRI-based radiomics nomogram for the preoperative prediction of deep myometrial invasion of FIGO stage I endometrial carcinoma. Med Phys.

[27] Qin L, Lai L, Wang H, Zhang Y, Qian X, He D (2022). Machine learning-based gray-level co-occurrence matrix (GLCM) models for predicting the depth of myometrial invasion in patients with stage i endometrial cancer. Cancer Manag Res.

[28] Otani S, Himoto Y, Nishio M, Fujimoto K, Moribata Y, Yakami M, Kurata Y, Hamanishi J, Ueda A, Minamiguchi S, Mandai M, Kido A (2022). Radiomic machine learning for pretreatment assessment of prognostic risk factors for endometrial cancer and its effects on radiologists' decisions of deep myometrial invasion. Magn Reson Imaging.

[29] Zhang K, Zhang Y, Fang X, Fang M, Shi B, Dong J, Qian L (2021). Nomograms of combining apparent diffusion coefficient value and radiomics for preoperative risk evaluation in endometrial carcinoma. Front Oncol.

[30] Stanzione A, Cuocolo R, Del Grosso R, Nardiello A, Romeo V, Travaglino A, Raffone A, Bifulco G, Zullo F, Insabato L, Maurea S, Mainenti PP (2021). Deep myometrial infiltration of endometrial cancer on MRI: a radiomics-powered machine learning pilot study. Acad Radiol.

[31] Rodríguez-Ortega A, Alegre A, Lago V, Carot-Sierra JM, Ten-Esteve A, Montoliu G, Domingo S, Alberich-Bayarri Á, Martí-Bonmatí L (2021). Machine learning-based integration of prognostic magnetic resonance imaging biomarkers for myometrial invasion stratification in endometrial cancer. J Magn Reson Imaging.

[32] Dong HC, Dong HK, Yu MH, Lin YH, Chang CC (2020). Using deep learning with convolutional neural network approach to identify the invasion depth of endometrial cancer in myometrium using MR images: a pilot study. Int J Environ Res Public Health.

[33] Chen X, Wang Y, Shen M, Yang B, Zhou Q, Yi Y, Liu W, Zhang G, Yang G, Zhang H (2020). Deep learning for the determination of myometrial invasion depth and automatic lesion identification in endometrial cancer MR imaging: a preliminary study in a single institution. Eur Radiol.

[34] Miccò M, Gui B, Russo L, Boldrini L, Lenkowicz J, Cicogna S, Cosentino F, Restaino G, Avesani G, Panico C, Moro F, Ciccarone F, Macchia G, Valentini V, Scambia G, Manfredi R, Fanfani F (2022). Preoperative tumor texture analysis on MRI for high-risk disease prediction in endometrial cancer: a hypothesis-generating study. J Pers Med.

[35] Ma C, Zhao Y, Song Q, Meng X, Xu Q, Tian S, Chen L, Wang N, Song Q, Lin L, Wang J, Liu A (2023). Multi-parametric MRI-based radiomics for preoperative prediction of multiple biological characteristics in endometrial cancer. Front Oncol.

[36] Lefebvre TL, Ueno Y, Dohan A, Chatterjee A, Vallières M, Winter-Reinhold E, Saif S, Levesque IR, Zeng XZ, Forghani R, Seuntjens J, Soyer P, Savadjiev P, Reinhold C (2022). Development and validation of multiparametric MRI-based radiomics models for preoperative risk stratification of endometrial cancer. Radiology.

[37] Deo RC (2015). Machine learning in medicine. Circulation.

[38] Greener JG, Kandathil SM, Moffat L, Jones DT (2022). A guide to machine learning for biologists. Nat Rev Mol Cell Biol.

