# Supplementary material for: Radiomics-Based Machine Learning for the Detection of Myometrial Invasion in Endometrial Cancer: Systematic Review and Meta-Analysis
Source: J Med Internet Res. 2025 Nov 27;27:e78809. doi: 10.2196/78809 (PMC12699251; doi:10.2196/78809)
Supplement: Multimedia Appendix 1 [file jmir_v27i1e78809_app1.docx]

**Supplement documents**


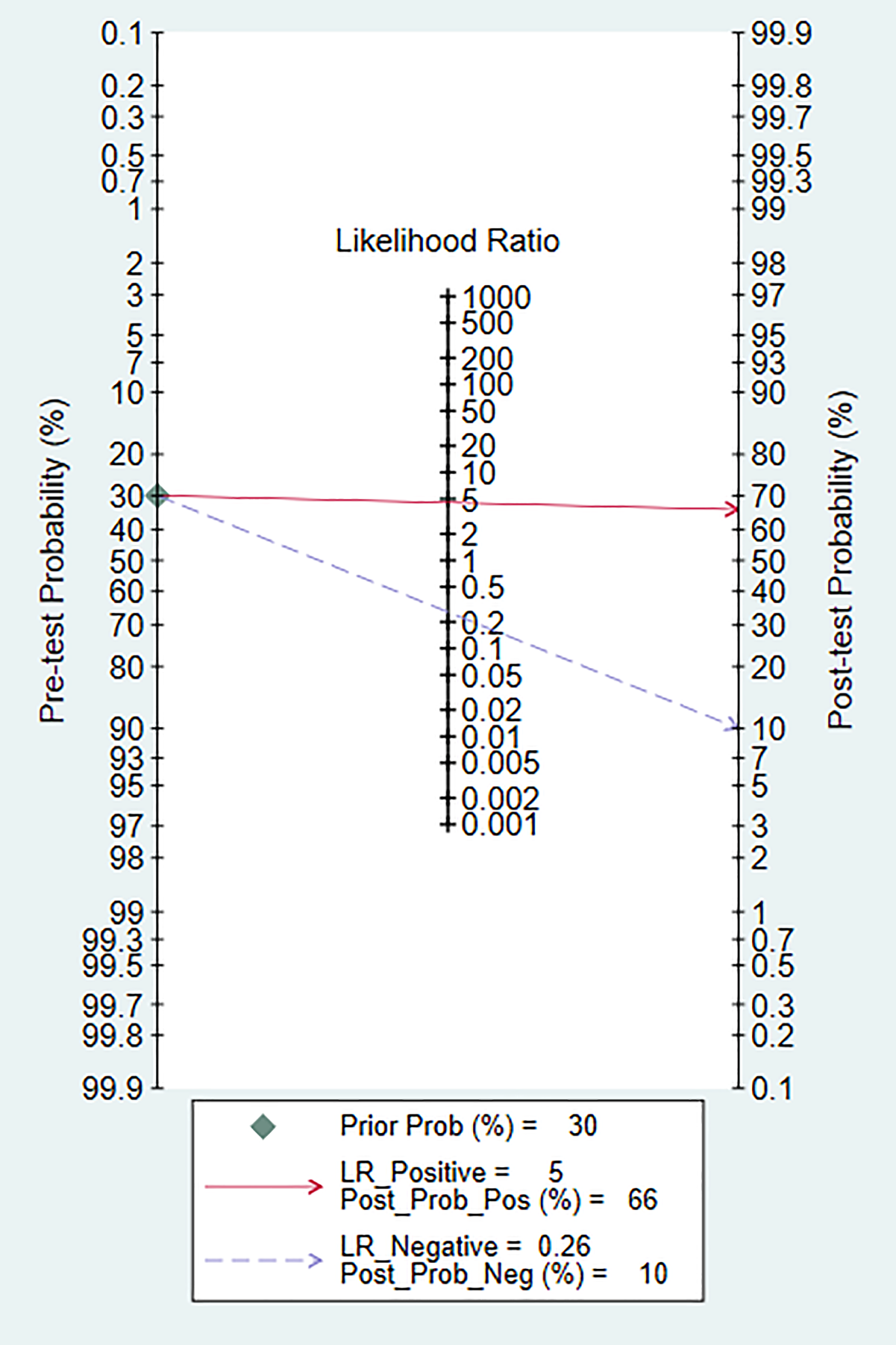


Figure S1. Fagan’s nomogram for post-test probability estimation of ML predictions.


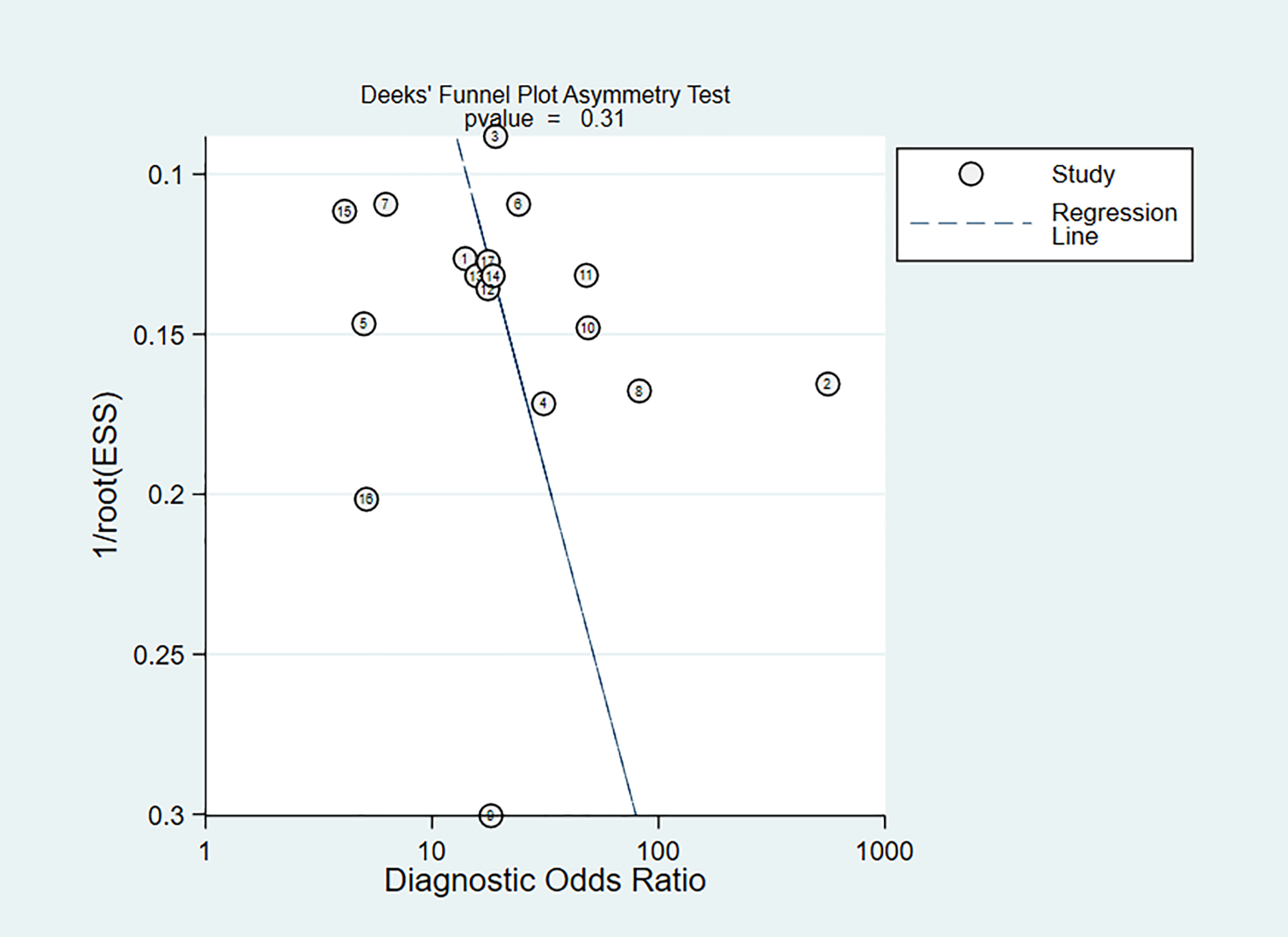


Figure S2. Deeks’ funnel plot for publication bias assessment in included ML studies.


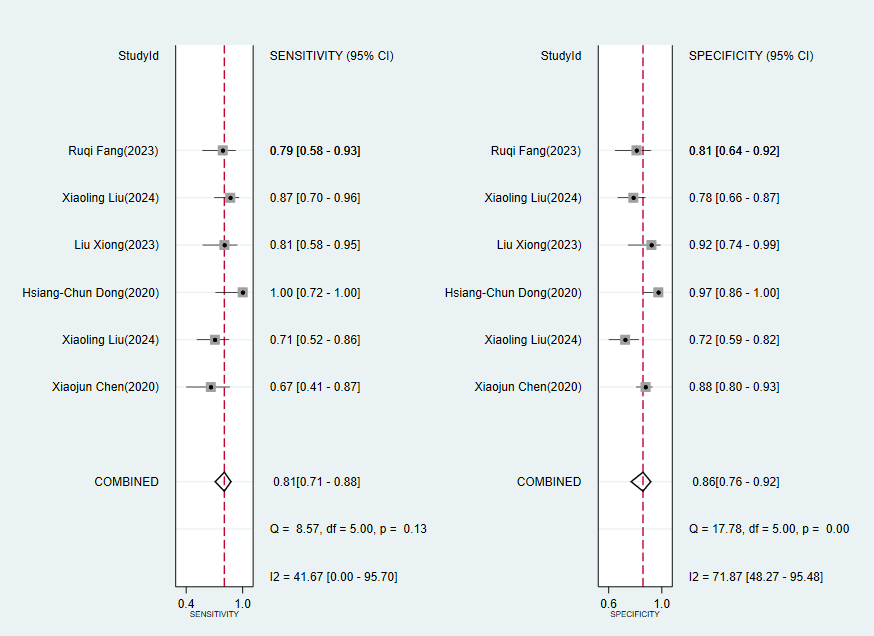
Figure S3. Forest plot of the sensitivity and specificity meta-analysis for DL in MI depth assessment in EC.


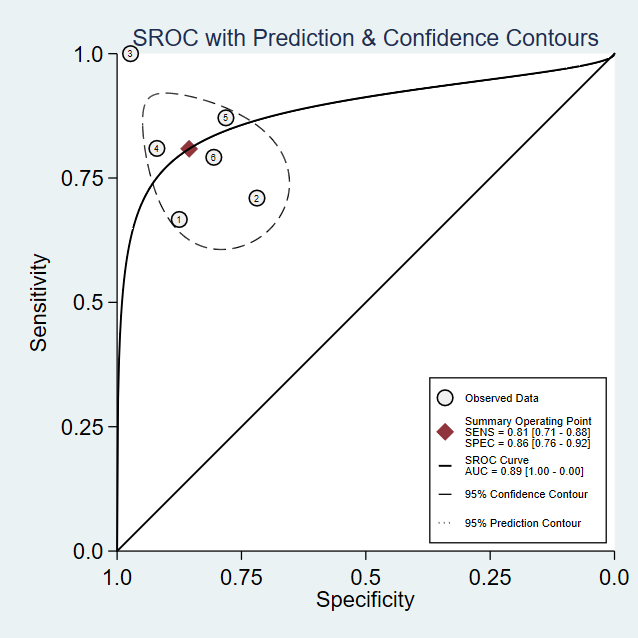


Figure S4. SROC curve for DL in MI depth assessment in EC.


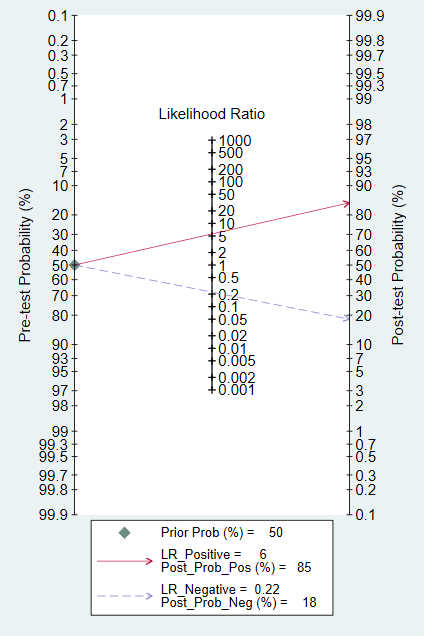


Figure S5. Fagan’s nomogram analysis of DL for the evaluation of MI in EC.


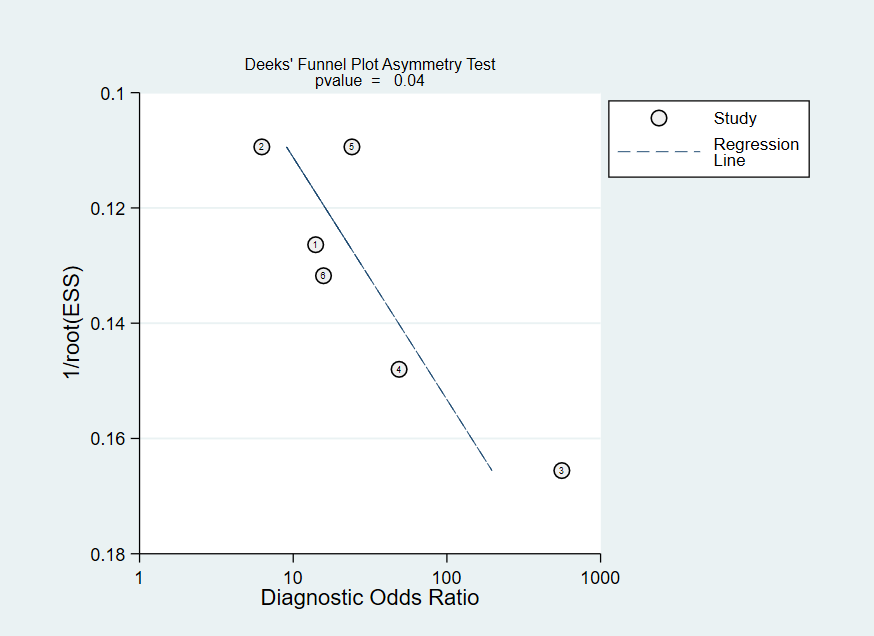


Figure S6. Deeks’ funnel plot for publication bias assessment in the DL meta-analysis for MI evaluation in EC.


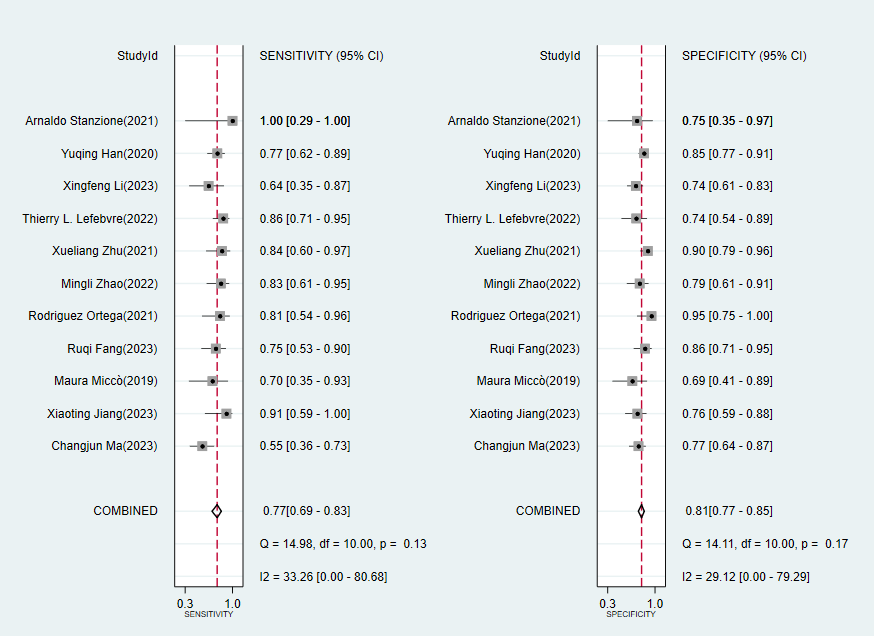


Figure S7. Forest plot of the meta-analysis of sensitivity and specificity for CML in MI depth assessment in EC.


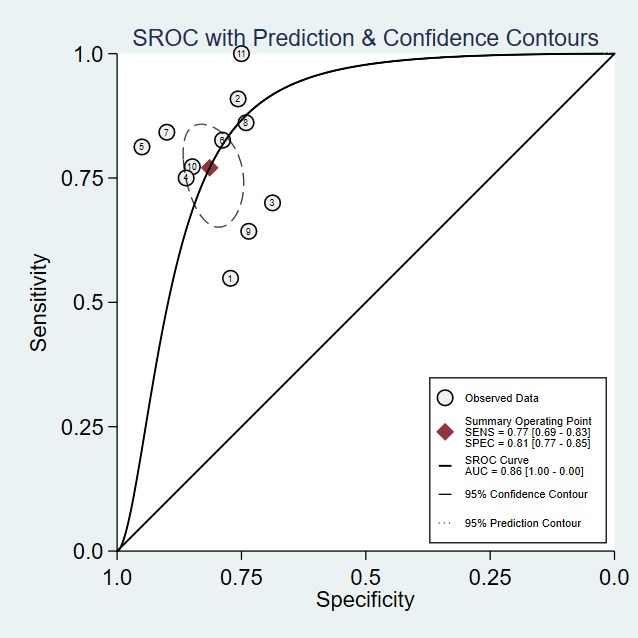


Figure S8. SROC curve for CML in evaluating MI in EC.


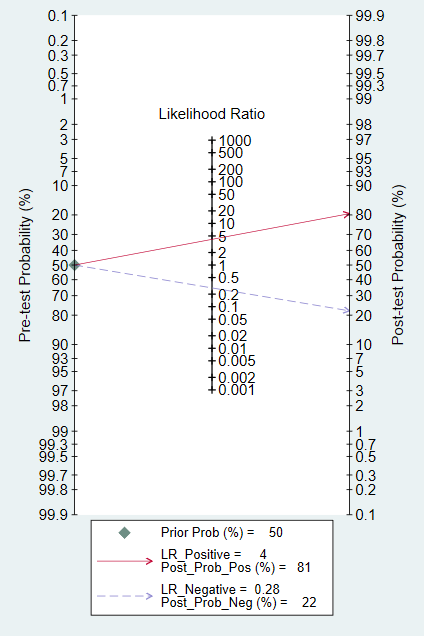


Figure S9. Fagan’s nomogram analysis of CML for assessing MI in EC.


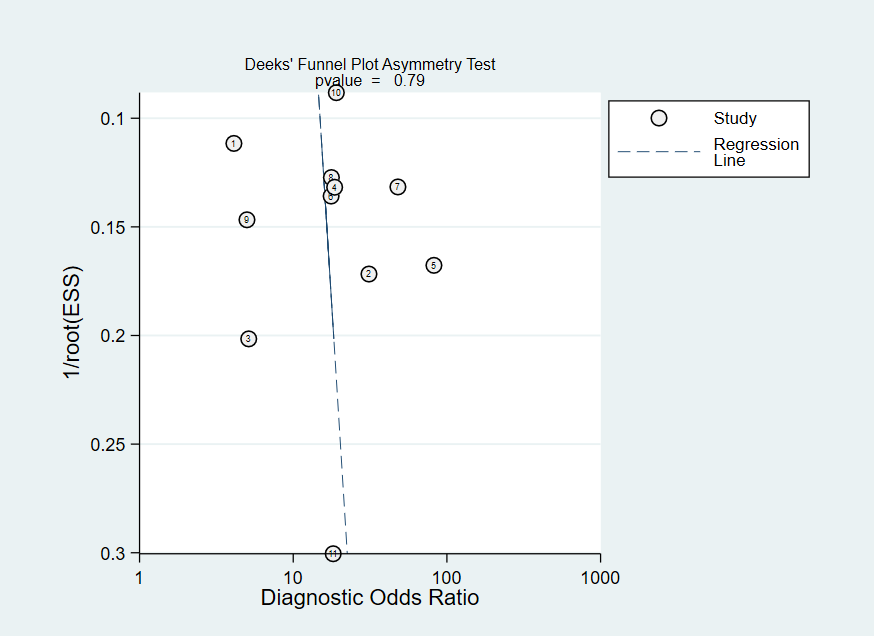


Figure S10. Deeks’ funnel plot for publication bias assessment in the CML meta-analysis for MI identification in EC.

Table S1 Search strategies

**1.Pubmed**

| Search number | Query | Results |
| --- | --- | --- |
| #1 | Endometrial Neoplasms[MeSH Terms] | 27,604 |
| #2 | ((((((((((((((((((Endometrial Neoplasms[Title/Abstract]) OR (Endometrial Neoplasm[Title/Abstract])) OR (Endometrial Carcinoma[Title/Abstract])) OR (Endometrial Carcinomas[Title/Abstract])) OR (Endometrial Cancer[Title/Abstract])) OR (Endometrial Cancers[Title/Abstract])) OR (Endometrium Cancer[Title/Abstract])) OR (Endometrium Carcinoma[Title/Abstract])) OR (Endometrium Carcinomas[Title/Abstract])) OR (Endometrium Cancers[Title/Abstract])) OR (endometrium tumor[Title/Abstract])) OR (endometrial tumor[Title/Abstract])) OR (endometrial tumour[Title/Abstract])) OR (endometrioma[Title/Abstract])) OR (endometrium tumour[Title/Abstract])) OR (Endometrioid Carcinoma[Title/Abstract])) OR (Endometrioid Carcinomas[Title/Abstract])) OR (Endometrioid Adenocarcinomas[Title/Abstract])) OR (Endometrioid Adenocarcinoma[Title/Abstract]) | 38,608 |
| #3 | (Endometrial Neoplasms[MeSH Terms]) OR (((((((((((((((((((Endometrial Neoplasms[Title/Abstract]) OR (Endometrial Neoplasm[Title/Abstract])) OR (Endometrial Carcinoma[Title/Abstract])) OR (Endometrial Carcinomas[Title/Abstract])) OR (Endometrial Cancer[Title/Abstract])) OR (Endometrial Cancers[Title/Abstract])) OR (Endometrium Cancer[Title/Abstract])) OR (Endometrium Carcinoma[Title/Abstract])) OR (Endometrium Carcinomas[Title/Abstract])) OR (Endometrium Cancers[Title/Abstract])) OR (endometrium tumor[Title/Abstract])) OR (endometrial tumor[Title/Abstract])) OR (endometrial tumour[Title/Abstract])) OR (endometrioma[Title/Abstract])) OR (endometrium tumour[Title/Abstract])) OR (Endometrioid Carcinoma[Title/Abstract])) OR (Endometrioid Carcinomas[Title/Abstract])) OR (Endometrioid Adenocarcinomas[Title/Abstract])) OR (Endometrioid Adenocarcinoma[Title/Abstract])) | 45,333 |
| #4 | machine learning[MeSH Terms] | 79,673 |
| #5 | (((((((((((((((((((((((((((((((machine learning[Title/Abstract]) OR (artificial intelligence[Title/Abstract])) OR (Transfer Learning[Title/Abstract])) OR (Deep learning[Title/Abstract])) OR (Ensemble Learning[Title/Abstract])) OR (random forest[Title/Abstract])) OR (neural network[Title/Abstract])) OR (neural networks[Title/Abstract])) OR (CNN[Title/Abstract])) OR (K-Nearest Neighbor[Title/Abstract])) OR (Support vector machine[Title/Abstract])) OR (SVM[Title/Abstract])) OR (Gradient Boosting Machine[Title/Abstract])) OR (Nomogram[Title/Abstract])) OR (XGBoost[Title/Abstract])) OR (Adaboost[Title/Abstract])) OR (LightGBM[Title/Abstract])) OR (CatBoost[Title/Abstract])) OR (Gradient Boosting[Title/Abstract])) OR (Decision tree[Title/Abstract])) OR (Regression Trees[Title/Abstract])) OR (ResNet[Title/Abstract])) OR (AlexNet[Title/Abstract])) OR (VGGNet[Title/Abstract])) OR (GoogLeNet[Title/Abstract])) OR (Naive Bayesian[Title/Abstract])) OR (Multilayer perceptron[Title/Abstract])) OR (Bayesian network[Title/Abstract])) OR (Radiomics[Title/Abstract])) OR (Radiomic[Title/Abstract])) OR (radiomics-based[Title/Abstract])) OR (Texture[Title/Abstract]) | 410,311 |
| #6 | (machine learning[MeSH Terms]) OR ((((((((((((((((((((((((((((((((machine learning[Title/Abstract]) OR (artificial intelligence[Title/Abstract])) OR (Transfer Learning[Title/Abstract])) OR (Deep learning[Title/Abstract])) OR (Ensemble Learning[Title/Abstract])) OR (random forest[Title/Abstract])) OR (neural network[Title/Abstract])) OR (neural networks[Title/Abstract])) OR (CNN[Title/Abstract])) OR (K-Nearest Neighbor[Title/Abstract])) OR (Support vector machine[Title/Abstract])) OR (SVM[Title/Abstract])) OR (Gradient Boosting Machine[Title/Abstract])) OR (Nomogram[Title/Abstract])) OR (XGBoost[Title/Abstract])) OR (Adaboost[Title/Abstract])) OR (LightGBM[Title/Abstract])) OR (CatBoost[Title/Abstract])) OR (Gradient Boosting[Title/Abstract])) OR (Decision tree[Title/Abstract])) OR (Regression Trees[Title/Abstract])) OR (ResNet[Title/Abstract])) OR (AlexNet[Title/Abstract])) OR (VGGNet[Title/Abstract])) OR (GoogLeNet[Title/Abstract])) OR (Naive Bayesian[Title/Abstract])) OR (Multilayer perceptron[Title/Abstract])) OR (Bayesian network[Title/Abstract])) OR (Radiomics[Title/Abstract])) OR (Radiomic[Title/Abstract])) OR (radiomics-based[Title/Abstract])) OR (Texture[Title/Abstract])) | 416,002 |
| #7 | ((Endometrial Neoplasms[MeSH Terms]) OR (((((((((((((((((((Endometrial Neoplasms[Title/Abstract]) OR (Endometrial Neoplasm[Title/Abstract])) OR (Endometrial Carcinoma[Title/Abstract])) OR (Endometrial Carcinomas[Title/Abstract])) OR (Endometrial Cancer[Title/Abstract])) OR (Endometrial Cancers[Title/Abstract])) OR (Endometrium Cancer[Title/Abstract])) OR (Endometrium Carcinoma[Title/Abstract])) OR (Endometrium Carcinomas[Title/Abstract])) OR (Endometrium Cancers[Title/Abstract])) OR (endometrium tumor[Title/Abstract])) OR (endometrial tumor[Title/Abstract])) OR (endometrial tumour[Title/Abstract])) OR (endometrioma[Title/Abstract])) OR (endometrium tumour[Title/Abstract])) OR (Endometrioid Carcinoma[Title/Abstract])) OR (Endometrioid Carcinomas[Title/Abstract])) OR (Endometrioid Adenocarcinomas[Title/Abstract])) OR (Endometrioid Adenocarcinoma[Title/Abstract]))) AND ((machine learning[MeSH Terms]) OR ((((((((((((((((((((((((((((((((machine learning[Title/Abstract]) OR (artificial intelligence[Title/Abstract])) OR (Transfer Learning[Title/Abstract])) OR (Deep learning[Title/Abstract])) OR (Ensemble Learning[Title/Abstract])) OR (random forest[Title/Abstract])) OR (neural network[Title/Abstract])) OR (neural networks[Title/Abstract])) OR (CNN[Title/Abstract])) OR (K-Nearest Neighbor[Title/Abstract])) OR (Support vector machine[Title/Abstract])) OR (SVM[Title/Abstract])) OR (Gradient Boosting Machine[Title/Abstract])) OR (Nomogram[Title/Abstract])) OR (XGBoost[Title/Abstract])) OR (Adaboost[Title/Abstract])) OR (LightGBM[Title/Abstract])) OR (CatBoost[Title/Abstract])) OR (Gradient Boosting[Title/Abstract])) OR (Decision tree[Title/Abstract])) OR (Regression Trees[Title/Abstract])) OR (ResNet[Title/Abstract])) OR (AlexNet[Title/Abstract])) OR (VGGNet[Title/Abstract])) OR (GoogLeNet[Title/Abstract])) OR (Naive Bayesian[Title/Abstract])) OR (Multilayer perceptron[Title/Abstract])) OR (Bayesian network[Title/Abstract])) OR (Radiomics[Title/Abstract])) OR (Radiomic[Title/Abstract])) OR (radiomics-based[Title/Abstract])) OR (Texture[Title/Abstract]))) | 722 |

**2.Cochrane**

| Search number | Query | Results |
| --- | --- | --- |
| #1 | MeSH descriptor: [Endometrial Neoplasms] explode all trees | 1236 |
| #2 | (Endometrial Neoplasm):ti,ab,kw OR (Endometrial Carcinoma):ti,ab,kw OR (Endometrial Carcinomas):ti,ab,kw OR (Endometrial Cancer):ti,ab,kw OR (Endometrial Cancers):ti,ab,kw | 3065 |
| #3 | (Endometrium Cancer):ti,ab,kw OR (Endometrium Carcinoma):ti,ab,kw OR (Endometrium Carcinomas):ti,ab,kw OR (Endometrium Cancers):ti,ab,kw OR (endometrium tumor):ti,ab,kw | 1879 |
| #4 | (endometrial tumor):ti,ab,kw OR (endometrial tumour):ti,ab,kw OR (endometrioma):ti,ab,kw OR (endometrium tumour):ti,ab,kw OR (Endometrioid Carcinoma):ti,ab,kw | 1567 |
| #5 | (Endometrioid Carcinomas):ti,ab,kw OR (Endometrioid Adenocarcinomas):ti,ab,kw OR (Endometrioid Adenocarcinoma):ti,ab,kw OR (Endometrial Neoplasms):ti,ab,kw | 1781 |
| #6 | #1 or #2 or #3 or #4 or #5 | 4201 |
| #7 | MeSH descriptor: [Machine Learning] explode all trees | 1035 |
| #8 | (artificial intelligence):ti,ab,kw OR (Transfer Learning):ti,ab,kw OR (Deep learning):ti,ab,kw OR (Ensemble Learning):ti,ab,kw OR (random forest):ti,ab,kw | 6360 |
| #9 | (neural network):ti,ab,kw OR (neural networks):ti,ab,kw OR (CNN):ti,ab,kw OR (K-Nearest Neighbor):ti,ab,kw OR (Support vector machine):ti,ab,kw | 4331 |
| #10 | (SVM):ti,ab,kw OR (Gradient Boosting Machine):ti,ab,kw OR (Nomogram):ti,ab,kw OR (XGBoost):ti,ab,kw OR (Adaboost):ti,ab,kw | 2377 |
| #11 | (LightGBM):ti,ab,kw OR (CatBoost):ti,ab,kw OR (Gradient Boosting):ti,ab,kw OR (Decision tree):ti,ab,kw OR (Regression Trees):ti,ab,kw | 1304 |
| #12 | (ResNet):ti,ab,kw OR (AlexNet):ti,ab,kw OR (VGGNet):ti,ab,kw OR (GoogLeNet):ti,ab,kw OR (Naive Bayesian):ti,ab,kw | 200 |
| #13 | (Multilayer perceptron):ti,ab,kw OR (Bayesian network):ti,ab,kw OR (Radiomics):ti,ab,kw OR (Radiomic):ti,ab,kw OR (radiomics-based):ti,ab,kw | 1227 |
| #14 | (machine learning):ti,ab,kw OR (Texture):ti,ab,kw | 5395 |
| #15 | #7 or #8 or #9 or #10 or #11 or #12 or #13 or #14 | 16845 |
| #16 | #6 and #15 | 37 |
| #17 |  |  |
| #18 |  |  |
| #19 |  |  |
| #20 |  |  |
| #21 |  |  |
| #22 |  |  |

**3.Embase**

| Search number | Query | Results |
| --- | --- | --- |
| #1 | 'endometrium tumor':ab,ti OR 'endometrial neoplasms':ab,ti OR 'endometrial neoplasm':ab,ti OR 'endometrial carcinoma':ab,ti OR 'endometrial carcinomas':ab,ti OR 'endometrial cancer':ab,ti OR 'endometrial cancers':ab,ti OR 'endometrium cancer':ab,ti OR 'endometrium carcinoma':ab,ti OR 'endometrium carcinomas':ab,ti OR 'endometrium cancers':ab,ti OR 'endometrial tumor':ab,ti OR 'endometrial tumour':ab,ti OR endometrioma:ab,ti OR 'endometrium tumour':ab,ti OR 'endometrioid carcinoma':ab,ti OR 'endometrioid carcinomas':ab,ti OR 'endometrioid adenocarcinomas':ab,ti OR 'endometrioid adenocarcinoma':ab,ti | 57071 |
| #2 | 'endometrium tumor'/exp | 92125 |
| #3 | #11 OR #12 | 98975 |
| #4 | 'machine learning'/exp | 527725 |
| #5 | 'machine learning':ab,ti OR 'artificial intelligence':ab,ti OR 'transfer learning':ab,ti OR 'deep learning':ab,ti OR 'ensemble learning':ab,ti OR 'random forest':ab,ti OR 'neural network':ab,ti OR 'neural networks':ab,ti OR cnn:ab,ti OR 'k-nearest neighbor':ab,ti OR 'support vector machine':ab,ti OR svm:ab,ti OR 'gradient boosting machine':ab,ti OR nomogram:ab,ti OR xgboost:ab,ti OR adaboost:ab,ti OR lightgbm:ab,ti OR catboost:ab,ti OR 'gradient boosting':ab,ti OR 'decision tree':ab,ti OR 'regression trees':ab,ti OR resnet:ab,ti OR alexnet:ab,ti OR vggnet:ab,ti OR googlenet:ab,ti OR 'naive bayesian':ab,ti OR 'multilayer perceptron':ab,ti OR 'bayesian network':ab,ti OR radiomics:ab,ti OR radiomic:ab,ti OR 'radiomics based':ab,ti OR texture:ab,ti | 521151 |
| #6 | #14 OR #15 | 773503 |
| #7 | #13 AND #16 | 1756 |

**4.Web of science**

| Search number | Query | Results |
| --- | --- | --- |
| #1 | Endometrial Neoplasms (Topic) OR Endometrial Neoplasm (Topic) OR Endometrial Carcinoma (Topic) OR Endometrial Carcinomas (Topic) OR Endometrial Cancer (Topic) OR Endometrial Cancers (Topic) OR Endometrium Cancer (Topic) OR Endometrium Carcinoma (Topic) OR Endometrium Carcinomas (Topic) OR Endometrium Cancers (Topic) OR endometrium tumor (Topic) OR endometrial tumor (Topic) OR endometrial tumour (Topic) OR endometrioma (Topic) OR endometrium tumour (Topic) OR Endometrioid Carcinoma (Topic) OR Endometrioid Carcinomas (Topic) OR Endometrioid Adenocarcinomas (Topic) OR Endometrioid Adenocarcinoma (Topic) | 66300 |
| #2 | machine learning (Topic) OR artificial intelligence (Topic) OR Transfer Learning (Topic) OR Deep learning (Topic) OR Ensemble Learning (Topic) OR random forest (Topic) OR neural network (Topic) OR neural networks (Topic) OR CNN (Topic) OR K-Nearest Neighbor (Topic) OR Support vector machine (Topic) OR SVM (Topic) OR Gradient Boosting Machine (Topic) OR Nomogram (Topic) OR XGBoost (Topic) OR Adaboost (Topic) OR LightGBM (Topic) OR CatBoost (Topic) OR Gradient Boosting (Topic) OR Decision tree (Topic) OR Regression Trees (Topic) OR ResNet (Topic) OR AlexNet (Topic) OR VGGNet (Topic) OR GoogLeNet (Topic) OR Naive Bayesian (Topic) OR Multilayer perceptron (Topic) OR Bayesian network (Topic) OR Radiomics (Topic) OR Radiomic (Topic) OR radiomics-based (Topic) OR Texture (Topic) | 2084217 |
| #3 | #1 AND #2 | 1010 |
